# Blockade of the PDGFR family together with SRC leads to diminished proliferation of colorectal cancer cells

**DOI:** 10.18632/oncotarget.1085

**Published:** 2013-06-30

**Authors:** Silke Kaulfuβ, Henning Seemann, Rovena Kampe, Julia Meyer, Ralf Dressel, Britta König, Jens-Gerd Scharf, Peter Burfeind

**Affiliations:** ^1^ Institute of Human Genetics, University Medical Center Göttingen, Germany; ^2^ Department of Cellular and Molecular Immunology, University Medical Center Göttingen, Germany; ^3^ Second Department of Internal Medicine, HELIOS Klinikum Erfurt GmbH, Germany

**Keywords:** colorectal cancer, PDGF receptor, c-KIT, SRC, cell proliferation, apoptosis, RNA interference, targeted therapy

## Abstract

Among the family of receptor tyrosine kinases (RTKs), platelet-derived growth factor receptor (PDGFR) has attracted increasing attention as a potential target of anti-tumor therapy in colorectal cancer (CRC). To study the function of PDGFRβ in CRC cell lines, SW480, DLD-1 and Caco-2 cells showing high PDGFRβ expression were used for receptor down-regulation by small interfering RNA (siRNA) and using the pharmacological inhibitor of PDGFRβ Ki11502. Blockade of PDGFRβ using both approaches led to moderate inhibition of proliferation and diminished activation of the downstream PI3K-signaling pathway in all three cell lines. Surprisingly, incubation with Ki11502 resulted in an arrest of SW480 cells in the G2 phase of the cell cycle, whereas the siRNA approach did not result in this effect. To address this difference, we analyzed the involvement of the PDGFRβ family member c-KIT in Ki11502 effectiveness, but siRNA and proliferation studies in SW480 and DLD-1 cells could not prove the involvement of c-KIT inactivation during Ki11502 treatment.

Hence, an RTK activation antibody array on SW480 cells led us to the identification of the non-receptor tyrosine kinase SRC, which is inactivated after Ki11502 treatment but not after the siRNA approach. Further studies using the SRC-specific inhibitor PP2 showed that SRC inhibition upon treatment with the inhibitor Ki11502 is responsible for the observed effects of Ki11502 in SW480 and DLD-1 CRC cells. In summary, our results demonstrate that the inhibition of PDGFRβ alone using siRNA has only moderate cellular effects in CRC cell lines; however, the multi-target inhibition of PDGFRβ, c-KIT and SRC, e.g., using Ki11502, represents a promising therapeutic intervention for the treatment of CRC.

## INTRODUCTION

Growth factors and their receptors play a significant role in the regulation of cancer growth, tumor angiogenesis and metastasis [1, 2], making these molecules attractive for targeted therapies. One of the approaches to target a growth factor receptor is the inhibition of epidermal growth factor receptor (EGFR) by inhibiting EGFR tyrosine kinase using small-molecule inhibitors or antibody-induced receptor blockade in different human cancers, including colorectal cancer (CRC). Blockade of EGFR signaling significantly inhibits tumor growth in animal models of colon cancer [3]. In clinical practice, using monoclonal antibodies to inhibit EGFR is now well established in the treatment of CRC [4].

Immunohistochemical studies on human colon cancer specimens revealed inter- and intratumoral heterogeneity for the expression of different receptor tyrosine kinases (RTKs), including EGFR, vascular endothelial growth factor receptor-2 (VEGFR2), and platelet-derived growth factor receptor β (PDGFRβ). This observation indicates that targeting a single RTK might not be sufficient for an optimal therapeutic effect [5]. We previously reported that the simultaneous blocking of two RTK pathways, i.e., the insulin-like growth factor receptor (IGF-IR) and EGFR pathways, produced more efficient therapeutic effects in colon cancer cell lines than the inhibition of a single pathway [6]. From these observations, it can be speculated that the therapeutic efficacy can be even further increased by inhibiting more than two RTK pathways.

PDGF and its receptor PDGFR represent a ligand-tyrosine kinase receptor system that is involved in different tumor-associated processes. PDGF binds to PDGFR and induces receptor dimerization and autophosphorylation, leading to the activation of intracellular signaling pathways [7]. PDGF plays a role in the autocrine growth stimulation of tumor cells, regulating tumor stroma fibroblast function and tumor angiogenesis. Different studies have indicated that PDGF and PDGFRβ are expressed by tumor cells [8, 9] as well as tumor-associated endothelial cells, pericytes, and other stromal cells in colon carcinomas [10-12], which are thought to provide a favorable microenvironment for the growth and survival of cancer cells [11, 13]. Furthermore, PDGFRβ expression levels are associated with angiogenesis, invasion and metastasis of colon cancer [10, 14, 15]. A recent study showed significantly increased PDGFRβ mRNA levels in locally advanced rectal tumors compared with the corresponding normal mucosa [16]. By contrast, in colon carcinoma cell lines expression of PDGFRβ at the mRNA level was lower. Imatinib mesylate, which inhibits phosphorylation of PDGFRβ, decreased the growth of primary cecal tumors and the incidence of liver metastasis in an orthotopic nude mouse model [17]. Some studies have elucidated the role of PDGF in colon cancer angiogenesis [12, 18].

Therefore, functional aspects of PDGFRβ expression were studied in CRC cell lines. In the present study, we showed that blockade of PDGFRβ alone using an siRNA approach does not lead to a sufficient reduction of proliferation. Instead, treatment with the PDGFRβ inhibitor Ki11502 resulted in a clear reduction of the proliferation and G2 cell cycle arrest of CRC cells, effects that were shown to be the result of the additional inhibition of c-KIT and SRC by Ki11502.

## RESULTS

### Expression of PDGF receptor α and β (PDGFRα and PDGFRβ) in CRC cells

**Figure 1:**
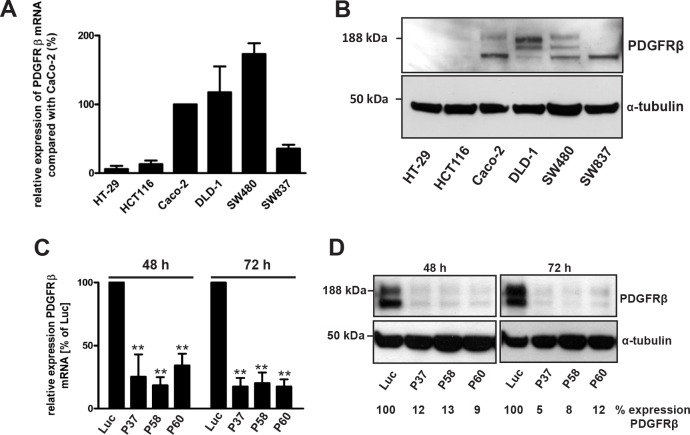
Relative mRNA expression of PDGFRβ in CRC cells and siRNA-mediated knockdown of PDGRβ in SW480 cells (A) Total RNA samples from different CRC cells were reverse-transcribed to cDNA and used to determine PDGFRβ mRNA expression using quantitative RT-PCR. The relative expression of PDGFRβ mRNA was expressed as the percentage of increase or decrease compared with Caco-2 cells after normalization against the expression of the housekeeping genes PBGD and TBP. Columns, mean; bars, SD. (B) Protein lysates (50 μg) isolated from different CRC cells were separated by SDS-PAGE, blotted, and analyzed by western blotting using PDGFRβ-specific antibodies. Equal loading of total protein was demonstrated by an α-tubulin-specific antibody. (C, D) SW480 cells were transiently transfected with different *PDGFRβ* gene-specific siRNAs (P37, P58, P60) or *luciferase* (Luc) gene-specific siRNA as a control. (C) Total RNA (1 μg) from transfected SW480 cells was reverse-transcribed to cDNA, and PDGFRβ mRNA expression was analyzed by quantitative RT-PCR. The expression of PDGFRβ mRNA was indicated as the percent decrease (mean ± SD) compared with Luc siRNA-transfected SW480 cells after normalization against expression of the two housekeeping genes PBGD and TBP. Statistically significant differences relative to Luc siRNA-transfected SW480 cells are indicated: **, P < 0.01 (Student's *t*-test). (D) Lysates of SW480 cells were size-fractionated by SDS-PAGE, blotted, and analyzed by western blotting with PDGFRβ-specific antibodies. Equal loading of total protein was ensured by stripping the membrane and re-probing with an α-tubulin-specific antibody. PDGFRβ-specific protein bands were densitometrically quantified. The relative densities of bands were indicated as the percent decrease compared with Luc siRNA-transfected SW480 cells (n = 3).

The expression levels of PDGFRα and β were evaluated in the CRC cell lines HT-29, HCT116, Caco-2, DLD-1, SW480 and SW837 by quantitative RT-PCR and western blotting (Fig. 1). The expression levels of PDGFRβ in HT-29, HCT116 and SW837 cells were significantly lower compared with Caco-2 cells (6 ± 5%, 13 ± 5% and 35 ± 13%, respectively). Higher expression levels of PDGFRβ were detectable in DLD-1 cells (117 ± 34%) and in SW480 cells (173 ± 31%) than in Caco-2 cells (Fig. 1A). Western blot analysis demonstrated PDGFRβ subunit-specific protein bands in Caco-2, DLD-1, SW480 and SW837 cells, respectively, whereas HT-29 and HCT116 cells failed to show any immunoreactivity against the PDGFRβ antibody (Fig. 1B). SW480 cells showing the highest PDGFRβ expression were chosen to study the role of PDGFRβ by siRNA-mediated receptor blockade or the RTK inhibitor Ki11502.

The expression of PDGFRα was undetectable in the CRC cell lines studied by quantitative RT-PCR or western blotting (data not shown).

### PDGFRβ blockade results in reduced cell proliferation

For silencing of PDGFRβ expression in SW480 cells, three different PDGFRβ-specific siRNAs (P37, P58 and P60) were used for transfection studies. SW480 cells transfected with siRNAs against the luciferase gene (Luc) were used as controls. The down-regulation of PDGFRβ mRNA expression in SW480 cells was determined by quantitative RT-PCR after siRNA transfection (Fig. 1C). The siRNA-mediated silencing of PDGFRβ mRNA expression was in the range of 10 to 20% of controls at 72 h after transfection (Fig. 1C).

In SW480 cells, western blot analysis revealed PDGFRβ-specific protein bands that were down-regulated in siRNA-transfected SW480 cells to approximately 9% to 13% of control levels of Luc-transfected (Luc) cells at 48 h after transfection and to approximately 5% to 12% at 72 h after transfection (Fig. 1D).

**Figure 2:**
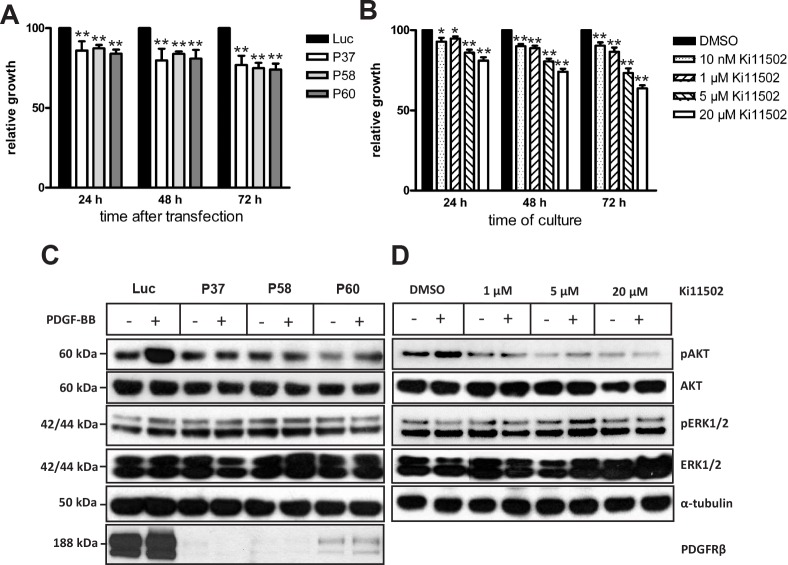
Inhibition of PDGFRβ by siRNA oligonucleotides or pharmacological inhibitor Ki11502 moderately decreases the proliferation and downstream signaling pathways (A) After transfection with PDGFRβ siRNA oligonucleotides (P37, P58, P60), SW480 cells were plated in a 96-well plate. After different time points of cultivation (24, 48 and 72 h), cell proliferation was determined using a non-radioactive assay, as described in the Materials and methods section. The results are expressed as the percent decrease (mean ± SD of three independent experiments) compared with Luc siRNA-transfected SW480 cells. Statistically significant differences relative to Luc siRNA-transfected SW480 cells are indicated: **, P < 0.01 (Student's *t*-test). (B) After starvation in serum-reduced medium for 24 h, SW480 cells were incubated in the presence or absence of the PDGFR tyrosine kinase inhibitor Ki11502 at the concentrations indicated. Control cultures were maintained in serum-reduced medium. All cells received the same amount of DMSO. After different periods of incubation (24, 48, and 72 h), cell proliferation was determined. The columns show mean percent decrease compared with SW480 cells treated only with the solvent (control) from three independent experiments; the bars show the SD. *, P < 0.05; **, P <0.01, statistically significant differences relative to control SW480 cells (Student's *t*-test). (C) SW480 cells were transfected with P37, P58, P60 or luciferase (Luc) siRNA. Seventy-two hours after transfection, serum-starved SW480 cells were incubated with PDGF-BB (10 ng/ml) or 10% FCS for 20 minutes and immediately subjected to detergent lysis. Proteins (15 μg) extracted from whole cell lysates were size-fractionated by SDS-PAGE and immunoblotted with antibodies against the PDGFRβ and phosphorylated forms of AKT and ERK1/2. Equal loading of proteins was demonstrated by immunoblotting with antibodies directed against the non-phosphorylated form of AKT and ERK1/2 as well as α-tubulin. The positions of the molecular weight standards are indicated on the left. (D) Serum-starved SW480 cells were cultivated in the presence of the PDGFR tyrosine kinase inhibitor Ki11502 for 24 h. Cells were then simultaneously incubated with PDGF-BB (10 ng/ml) or without PDGF-BB for 20 minutes and were immediately proceeded for detergent lysis and western blot analysis against the phosphorylated and non-phosphorylated forms of AKT and ERK1/2 as outlined.

As a second approach, the pharmacological PDGFRβ tyrosine kinase inhibitor Ki11502 was used. This inhibitor was tested to be exclusively specific for PDGFRβ in leukemia and vascular smooth muscle cells (19, 26), not targeting PDGFRα and c-KIT. To study whether blockade of PDGFRβ in SW480 cells was associated with alterations in cell proliferation and apoptosis, SW480 cells were transfected with PDGFRβ-specific siRNAs or cultivated in the presence of the inhibitor Ki11502. The siRNA-dependent blockade of PDGFRβ was associated with a moderate but significant inhibition of cell proliferation at 24 and 48 h after transfection compared with Luc-siRNA-transfected SW480 cells (Fig. 2A). Seventy-two hours after transfection, PDGFRβ siRNA transfection was associated with a reduction in cell proliferation to approximately 74% to 77% of the levels observed in Luc-transfected SW480 cells. Treatment of SW480 cells with Ki11502 resulted in the time- and dose-dependent inhibition of cell proliferation (Fig. 2B). Maximal effects were observed after incubating SW480 cells with 20 μM Ki11502 for 72 h, showing a reduction in cell proliferation to approximately 64% of the levels observed in DMSO-treated control cells (Fig. 2B).

The siRNA-mediated down-regulation of PDGFRβ was associated with a moderate increase of up to 5% dead cells compared with Luc-transfected cells (Suppl. Fig. 1A). Similarly, the PDGFR tyrosine kinase inhibitor Ki11502 only marginally stimulated the increase of dead cells at all concentrations tested (Suppl. Fig. 1B).

### PDGFRβ down-regulation is associated with altered signaling

We next evaluated whether blockade of PDGFRβ by either siRNA or the inhibitor Ki11502 affected the phosphorylation of downstream signaling proteins such as AKT and ERK1/2. Treatment of SW480 cells with PDGF-BB for 20 minutes stimulated the phosphorylation of AKT (Ser473), but no activation of ERK1/2 was detected (Figs. 2C, 2D). In SW480 cells transfected with the PDGFRβ-specific siRNAs P37, P58 and P60, the PDGF-BB-mediated phosphorylation of AKT was abrogated (Fig. 2C). The PDGFRβ inhibitor Ki11502 reduced the PDGF-BB-induced phosphorylation of AKT upon treatment of cells with 1 μM Ki11502, showing a significant inhibition of AKT phosphorylation at a concentration of 5 μM. There was no detectable change of ERK1/2 phosphorylation (Fig. 2D).

### Effect of PDGFRβ blockade on the cell cycle

**FIGURE 3:**
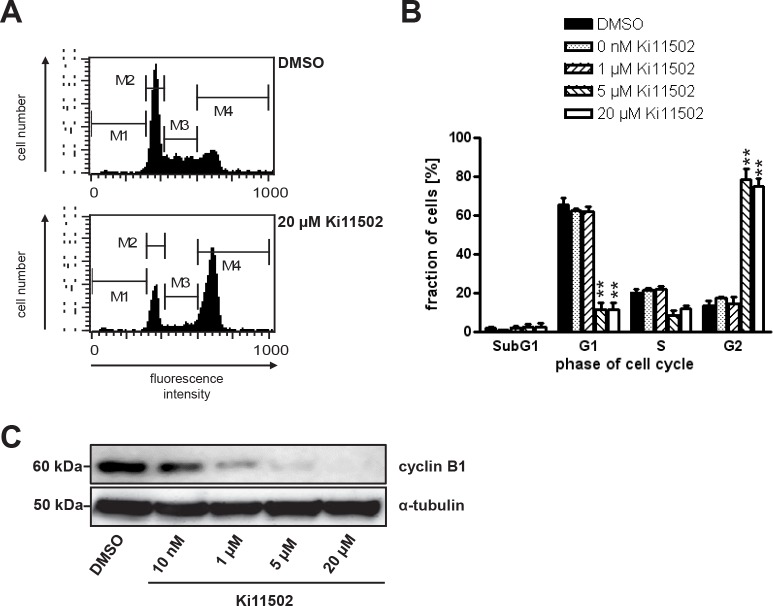
Effect of PDGFR blockade on the cell cycle (a, B) SW480 cells were treated with the indicated concentrations of Ki11502 for 72 h. Cells were fixed and stained with propidium iodide and subjected to flow cytometry. (A) Representative examples of blots for control-treated cells (DMSO) and cells treated with 20 μM Ki11502. (B) Three independent experiments were performed and summarized. Bars, SD; **, P <0.01, statistically significant differences relative to SW480 cells treated with DMSO (Student's *t*-test). (C) Protein lysates of SW480 cells treated with the indicated concentrations of Ki11502 for 72 h were subjected to SDS-PAGE, blotted and incubated with antibody against cyclin B1. Antibody against α-tubulin was used to show equal sample loading.

To address whether the decrease of cell proliferation after inhibition of PDGFRβ was linked to alterations in cell cycle progression, SW480 cells either transfected with PDGFRβ-specific siRNAs or treated with the PDGFR tyrosine kinase inhibitor Ki11502 were subjected to flow cytometric analysis (Fig. 3). These experiments demonstrated that in DMSO-treated control cells, the major fraction of cells were in the G1 phase (65.2 ± 7.7%), whereas only a minor portion of cells were in the S or G2 phase, respectively. Treatment of SW480 cells with 20 μM Ki11502 resulted in a reduction of cells in the G1 phase (11.4 ± 7.6%), whereas most cells were arrested in the G2 phase (74.8 ± 9.1%) (Figs. 3A, 3B).

Unexpectedly, the down-regulation of PDGFRβ in SW480 cells using the siRNA approach did not result in G2 arrest (Suppl. Fig. 2). Cell cycle analyses did not reveal any detectable changes using flow cytometry.

To investigate which cell cycle proteins might be deregulated after inhibitor-mediated PDGFRβ blockade, western blot analyses were performed. These experiments demonstrated that the cell cycle protein cyclin B1, which is necessary to transit the G2/M checkpoint and thus initiate mitosis, is clearly down-regulated in SW480 cells after treatment with Ki11502 in a dose-dependent manner (Fig. 3C).

### Does the additional inhibition of c-Kit result in the difference observed between the siRNA- and inhibitor-mediated blockades of PDGFRβ?

**FIGURE 4:**
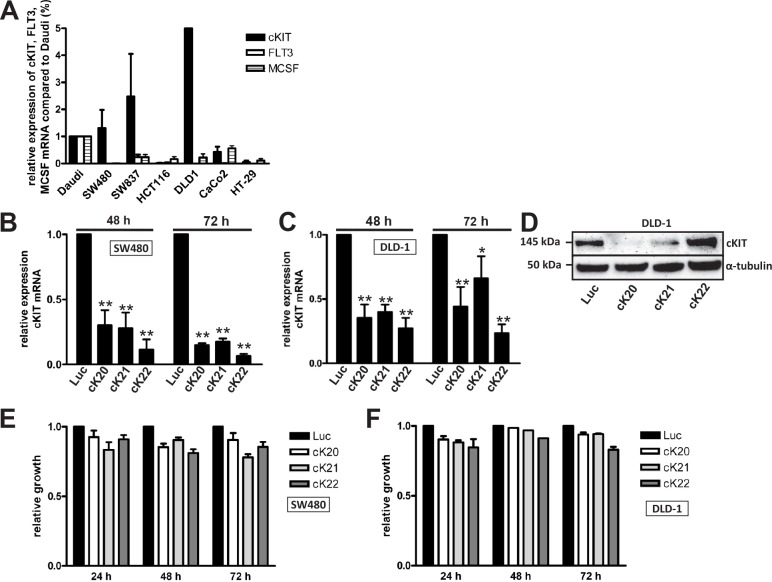
Expression and influence of the PDGFRβ family member c-KIT on cell proliferation (A) A panel of CRC cell lines was screened for the expression of c-KIT, FLT3 and MCSF using quantitative RT-PCR. Data from three independent experiments are presented relative to the control cells Daudi. (B-F) c-KIT expression was down-regulated in SW480 and DLD-1 cells using siRNAs (cK20, cK21 and cK22). As a control, cells were transfected with siRNA against the luciferase gene. Quantitative RT-PCR (B, C) and western blot analyses (D) 48 and 72 h after transfection showed decreased expression levels of c-KIT. (E, F) Decreased proliferation after c-KIT knockdown in SW480 (E) and DLD-1 (F) cells. Statistically significant differences relative to Luc siRNA-transfected CRC cells are indicated: *, P < 0.05; **, P < 0.01 (Student's *t*-test).

Because c-KIT, FLT3 and MSCF, and PDGFRβ belong all to the same RTK family, we hypothesized that Ki11502 is not as specific for PDGFRβ as expected. Therefore, we first screened different CRC cell lines for c-KIT, FLT3 and MSCF expression in comparison to the Burkitt lymphoma cell line Daudi (Fig. 4A). DLD-1 cells showed very strong c-KIT expression; however, Caco-2 cells showed no c-KIT expression, and SW480 cells displayed moderate to weak expression. All three cell lines expressed PDGFRβ at the protein and mRNA levels. FLT3 and MSCF were only hardly expressed in all cell lines tested.

To analyze the influence of the Ki11502 inhibitor on c-KIT activity, we treated DLD-1 and Caco-2 cells with Ki11502 or down-regulated the expression of PDGFRβ using the established siRNA approach (Suppl. Figs. 3A, 3B) and measured the capacity of cell proliferation and cell cycle distribution. DLD-1 cells showed a strong decrease of proliferation (>60%) 72 h after the inhibition of PDGFRβ using Ki11502. However, only a moderate inhibition of proliferation was observed when using the RNAi approach (Suppl. Figs. 3C, 3E), although the down-regulation was verified using qPCR and western blot analyses (Suppl. Figs. 3A, 3B). Using flow cytometry analyses, we also observed an increase of cells in the SubG1 phase, indicating that these cells might undergo apoptosis (Suppl. Fig. 4A). However, Caco-2 cells only moderately responded to either type of PDGFRβ inhibition, showing only a 20% reduction in proliferation (Suppl. Figs. 3D, 3F). There was an increase of cells in the G2 phase after Ki11502 inhibitor treatment (Suppl. Fig. 4B).

We then down-regulated the expression of c-KIT in SW480 (Figs. 4B, 4E) and DLD-1 (Figs. 4C, 4D, and 4F) cells (high expression of c-KIT) using three different siRNAs (Figs. 4B, 4C, and 4D). Although c-KIT expression was diminished in both cell lines, only a moderate inhibition of proliferation was observed (Figs. 4E, 4F). These results suggest that c-KIT inhibition is not the underlying cause of the effectiveness of Ki11502 in SW480 and DLD-1 cells.

### SRC is inhibited by Ki11502 and contributes to the inhibition of proliferation in CRC cell lines

**FIGURE 5:**
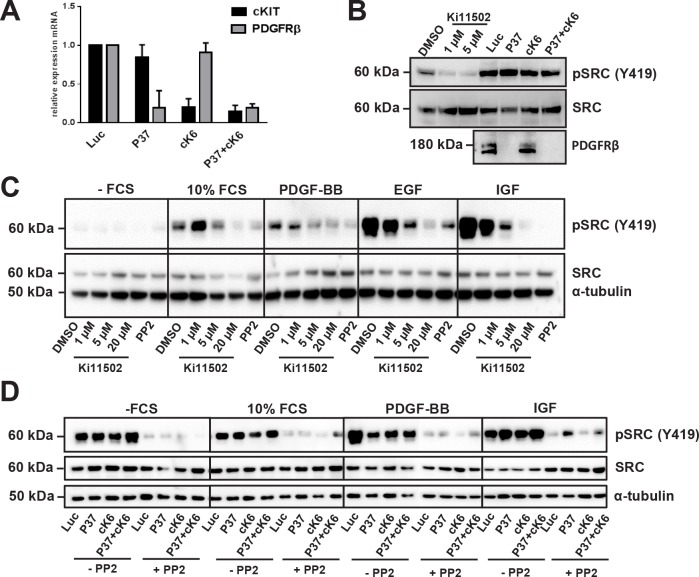
SRC activation after PDGFRβ inhibition using siRNA or the small-molecule inhibitor Ki11502 in SW480 cells (A) Quantitative RT-PCR to confirm the down-regulation of PDGFRβ and c-KIT 72 h after transfection with the appropriate siRNAs against PDGFRβ (P37) and c-KIT (cK6) in SW480 cells. (B) Verification of results obtained using RTK activation array analysis. SW480 cells were treated either with 1 and 5 μM Ki11502 and vehicle control or transfected with siRNA against PDGFRβ (P37), c-KIT (cK6) or both (P37+cK6). After 48 h, cells were serum-starved overnight and stimulated with 10% FCS for 10 minutes followed by protein isolation. SRC activation was detected using an antibody against phosphorylated Tyr416. Down-regulation was checked using a PDGFRβ-specific antibody. (C) SW480 cells were treated with Ki11502 (1, 5 and 20 μM) or with 10 μM of the SRC inhibitor PP2 for 48 h. After serum-starvation, cells were stimulated with 10% FCS, PDGF-BB, EGF and IGF for 10 minutes. (D) SW480 cells were transfected with siRNA against PDGFRβ (P37), c-KIT (cK6) or both (P37+cK6) compared with control-transfected cells (Luc) and were then treated with or without 10 μM PP2 for 48 h. After serum-starvation, cells were stimulated with 10% FCS, PDGF-BB, EGF and IGF-I for 10 minutes, whereas inhibitor treatment resulted in the reduction of SRC activation (B, C), and the down-regulation of PDGFRβ, c-KIT or both did not change the SRC activation pattern (D).

**FIGURE 6:**
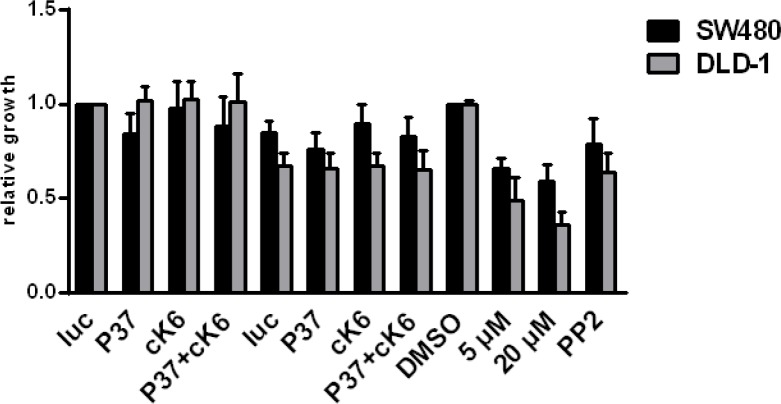
Influence of SRC inhibition on the proliferation capacity of CRC cells DLD-1 and SW480 cells were transfected with siRNAs against PDGFRβ (P37), c-KIT (cK6) or both (P37+cK6) in the presence or absence of 10 μM of the SRC inhibitor PP2. Proliferation was determined 72 h after transfection. Treatment with PP2 in addition to the down-regulation of PDGFRβ and c-KIT results in a stronger reduction of proliferation, showing the highest effects in DLD-1 cells compared with the effects of PP2 single treatments. Treatment with Ki11502 still demonstrates stronger effects than the combination strategy of siRNA and PP2. Statistically significant differences relative to Luc siRNA-transfected and DMSO treated CRC cells are indicated, respectively: *, P < 0.05; **, P < 0.01 (Student's *t*-test).

To determine whether other RTKs might be influenced by treating CRC cells with the Ki11502 inhibitor, we performed an RTK activity assay. Using this antibody-based approach, we screened for the activity of several RTKs and their downstream signaling components after the treatment of SW480 cells with siRNAs against PDGFRβ, c-KIT, and the combination of both (Fig. 5A), as well as with two concentrations of Ki11502 (1 and 5 μM). We performed this array twice with independent biological samples. In both experiments, the non-receptor tyrosine kinase SRC showed a special activation pattern: whereas the downregulation of PDGFRβ, c-KIT or the combination using the siRNA approach did not result in a change of SRC phosphorylation, Ki11502 treatment inhibited the activation of SRC after stimulation with 10% FCS (data not shown). We verified the array results using the same biological samples by western blot analysis (Fig. 5B). Furthermore, the treatment of SW480 and DLD-1 cells with Ki11502 resulted in a reduction in SRC activation similar to that observed after treating these cells with the SRC-specific inhibitor PP2. No difference was observed among the stimuli used (FCS, IGF-I, EGF or PDGF-BB; Fig. 5C, Suppl. Fig. 5A). In contrast, the down-regulation of the receptors PDGFRβ, c-KIT or both did not change the activation of SRC compared with the control-transfected cells (Fig. 5D, Suppl. Fig. 5B). Additional treatment of both cell lines showing reduced receptor expression with the SRC-specific inhibitor PP2 reduced the phosphorylation of SRC and, therefore, the activity of this tyrosine kinase. We then analyzed the influence of SRC inhibition on the proliferation capacity of SW480 and DLD-1 cells. As depicted in Fig. 6, the down-regulation of PDGFRβ and c-KIT alone resulted in a moderate decrease in cell proliferation, whereas adding the SRC-specific inhibitor PP2 diminished the proliferation capacity to the level observed after using the PDGFRβ inhibitor Ki11502. DLD-1 cells responded more strongly to the treatment with PP2, and proliferation was decreased to levels observed for the Ki11502 inhibitor. Taken together, these results support the hypothesis that the PDGFRβ inhibitor Ki11502 inhibits SRC, thus resulting in the cellular effects of decreased proliferation and cell cycle arrest, both of which could not be achieved through the downregulation of PDGFRβ using the siRNA approach.

## DISCUSSION

In the present study, a subset of different CRC cell lines was analyzed for the expression of the α- and β-subunits of PDGFR. The highest levels of PDGFRβ were detectable in SW480 cells, whereas PDGFRα was below the limits of detection in all CRC lines analyzed. Therefore, SW480 cells were firstly used to investigate the effect of the down-regulation of PDGFRβ expression using siRNA technology and the pharmacological inhibitor of PDGFRβ tyrosine kinase Ki11502, which was described to be specific for PDGFRβ. After transfection with PDGFRβ-specific siRNAs, the mRNA and protein levels of PDGFRβ in SW480 cells were significantly decreased. The siRNA-mediated knockdown of the PDGFRβ inhibited the proliferation of SW480 cells, whereas its effect on cytotoxicity was only marginal. The pharmacological inhibition of PDGFRβ by the inhibitor Ki11502 resulted in a dose-dependent inhibition of proliferation and showed no significant effect on the cytotoxicity of SW480 cells, even when cells were treated with high concentrations of Ki11502 (>5 μM). The activation of PDGFRβ by PDGF-BB induced the phosphorylation of AKT, which is a known downstream component of the signaling pathways of PDGFRβ. Receptor blockade via siRNA oligonucleotides directed against PDGFRβ and Ki11502 inhibited the phosphorylation of AKT, supporting the convergence between the siRNA approach and small-molecule inhibitor treatment. In contrast, after treatment with higher concentrations of Ki11502, SW480 cells were arrested in the G2 phase of the cell cycle. This effect was not visible using the siRNA approach, leading us to ask whether the small-molecule inhibitor Ki11502 was not as specific as expected from the literature [19]. Due to the high expression of c-KIT in DLD-1 cells, which responded to the treatment with Ki11502 to a higher extent than SW480 cells, we speculated that c-KIT inhibition by Ki11502 might be the underlying cause of the difference between the siRNA approach and small-molecule inhibitor treatment. This hypothesis was supported by the finding that Caco-2 cells, which express c-KIT beyond the limits of detection, did not present with reduced proliferation after treatment with Ki11502. However, the down-regulation of c-KIT alone or in combination with PDGFRβ in SW480 and DLD-1 cells did not result in the observed effects mediated by Ki11502, indicating that additional c-KIT inhibition is unlikely to be an underlying molecular cause of Ki11502 inhibition.

Therefore, we performed antibody-based screening of RTKs and their signaling activation. Using this approach, we determined that the tyrosine kinase SRC was inactivated after Ki11502 treatment in CRC cells, whereas there was no influence on or even an increase in SRC activation after the down-regulation of PDGFRβ, c-KIT or both using siRNAs. SRC plays an integral role in multiple cellular processes through its interaction with structural and signaling proteins through its SH2 and SH3 domains, including invasion, migration, proliferation, angiogenesis, and apoptosis. SRC is activated by several processes, including binding to growth factor receptors and integrins. Because SRC activation has been implicated in a large percentage of common solid tumor types, SRC has become a recent target for drug therapy (reviewed in [20]). In CRC, SRC deregulation was found in 80% of cases, with increasing SRC activity along the adenoma-carcinoma sequence from normal mucosa and colonic polyps to distant liver metastases [21, 22]. Hence, increased SRC activity represents an independent indicator of poor clinical prognosis in all stages of colon cancer [23].

SRC plays a critical role in the mediation of signaling through PDGFR [24, 25]. Therefore, we cannot fully rule out that the reduced activation of SRC after treatment with the inhibitor Ki11502 is due to the inhibition of PDGFR signaling, whereas residual active PDGFRβ in PDGFRβ knockdown cells can still activate downstream signaling via SRC. Nevertheless, in the latter case, we expected at least the minimal inhibition of SRC activation, which was completely absent in our experiments. This result supports our hypothesis that Ki11502 does additionally inhibit SRC activation. Notably, in the original publication, the specificity of Ki11502 was verified by screening the activation status of a number of RTKs; however, the influence of Ki11502 on the activation of SRC was not investigated [19]. Subsequent publications considering Ki11502 also did not include SRC in their portfolio [26, 27].

Our results also continue the discussion of whether the down-regulation of certain proteins using siRNA or knockout approaches is the correct methodology to validate the specificity of small-molecule inhibitors [28]. On the one hand, an exclusive examination of our siRNA studies against PDGFRβ would have resulted in the findings that PDGFRβ plays a minor role in CRC and that targeting this RTK will not lead to an improvement of therapy options for patients. On the other hand, focusing only on small-molecule inhibitor studies would have clearly indicated the need for further studies on the influence of PDGFRβ in CRC and in the continued use of this therapy option, not knowing that the observed effects are due to the side inhibition of additional kinases. Only a comparison of the results of both studies led to our hypothesis that PDGFRβ alone is not essential in the progression of CRC. However, effective blockade of PDGFRβ together with its family member c-KIT and the downstream signaling molecule SRC might be a promising therapy approach for CRC in the future.

The blockade of multiple RTKs as a molecular targeted therapy option is not a new issue. We [6] and others [29] have already shown that the inhibition of more than one RTK is more effective in the treatment of various solid tumors than is a single-RTK blockade. Three different PDGFR tyrosine kinase inhibitors are currently under clinical investigation for the treatment of CRC, i.e., imatinib mesylate, sunitinib, and sorafenib, which all inhibit, in addition to PDGFR, other RTKs such as SCF, VEGF and KIT or the Raf cascade, respectively [30, 31]. Imatinib mesylate has shown anti-tumor activity in human CRC cells in preclinical and phase II studies [32]. A phase I/II study of XELOX in combination with bevacizumab and imatinib in first-line metastatic CRC (mCRC) is ongoing. Phase II/III studies are evaluating the activity of sunitinib and sorafenib in patients with mCRC. Sorafenib has shown clinical activity in the initial phase I study. A phase II study of sorafenib and capecitabine (SorCape) in previously treated metastatic colorectal cancer (mCRC) (NCT01471353) showed promising results, with a preliminary PFS of 4.1 months [33]. In addition, several clinical trials have tested the activity of sorafenib in combination with oxaliplatin- and irinotecan-based chemotherapy and with other targeted therapies, such as cetuximab, in mCRC patients. Novel multi-targeted kinase inhibitors have been developed, e.g., ABT-348, a new ATP-competitive multi-targeted kinase inhibitor against Aurora, VEGF/PDGFR and SRC with nanomolar potency [34]. The first *in vitro* and xenograft studies demonstrated high efficiency in solid tumors and hematological malignancies for ABT-348 and are now awaiting *in vivo* approval.

In summary, the present study shows that the inhibition of PDGFRβ alone has no effective influence in CRC cells, but blockade of PDGFRβ, c-KIT and SRC using the small-molecule inhibitor Ki11502 decreases the proliferation capacity of CRC cells, supporting ongoing studies for the implementation of such multitarget treatments in clinical issues.

## MATERIALS AND METHODS

### Materials

Chemicals were reagent grade and commercially obtained as mentioned: recombinant human IGF-I (GroPep, Adelaide, Australia); the PDGFR tyrosine kinase inhibitor Ki11502 (Merck Millipore, Darmstadt, Germany), PP2, recombinant PDGF-BB, propidium-iodide (both obtained from Sigma-Aldrich, Munich, Germany), recombinant EGF (Cell Signaling, Beverly, MA, USA), protease inhibitors (Serva, Heidelberg, Germany), phosphatase inhibitors (Roche, Mannheim, Germany), and RNase A (Applichem, Darmstadt, Germany).

### Antibodies

The following antibodies and sera were purchased from commercial sources as indicated: mouse monoclonal antibody directed against c-Kit (Ab81) and rabbit monoclonal antibodies directed against phospho-ERK1/2 (Thr202/Tyr204 (D13.13.4E)), ERK1/2 (137F5), phospho-Akt (Ser473 (D9E)), Akt (C67E7), PDGFRβ (28E1), phospho-SRC (Tyr416 (D49G4)), SRC (32G6) (all from Cell Signaling), mouse monoclonal antibody raised against α-tubulin (Sigma), peroxidase-conjugated AffiniPure rabbit anti-mouse IgG and goat anti-rabbit IgG (Dianova, Hamburg, Germany).

### Cell lines and cell culture

The human colon cancer cell lines SW480, Caco-2 and DLD-1 were obtained from the American Type Culture Collection (ATCC, Rockville, MD, USA) and approved for cell line contamination using STR-profiling. Caco-2 cells were maintained in Minimum Essential Medium (MEM) supplemented with 20% fetal calf serum (FCS), and DLD-1 and SW480 cells were maintained in RPMI 1640 supplemented with 10% FCS and 1.2% penicillin/streptomycin (PAN-Systems) at 37°C and 5% CO_2_ in humidified air. The medium was changed three times per week, and cells were passaged using trypsin/EDTA.

### Treatment of CRC cells

Before addition of stimuli, cells were allowed to grow until 70% confluency and were then washed with PBS. All cultures were maintained under serum-reduced conditions by addition of the specified media without FCS overnight, and then incubated with or without growth factors (1 nM IGF-I, 100 ng/ml EGF, 10 ng/ml PDGF-BB) for 10 minutes at 37°C. The cells were washed with cold PBS and immediately processed for RNA isolation or protein extraction. For treatment with Ki11502 and PP2, cells were incubated in the presence of the inhibitor for 48 h followed by serum starvation overnight. Growth factors were added the next day for 10 minutes, followed by protein isolation.

### Protein extraction and Western blot analysis

Cell lysates were prepared using lysis buffer containing 50 mM Tris-HCl (pH 7.4), 150 mM NaCl, 1 mM EDTA, 1% NP-40, 0.25% sodium deoxycholate, protease inhibitors (complete mini) and PhosStop (both Roche). Protein concentration was determined using the Bradford assay (Nanoquant, Carl Roth, Karlsruhe, Germany). Aliquots of 10 to 50 μg of total cell lysates were boiled and denatured in sample buffer containing SDS and dithiothreitol (DTT; Invitrogen) followed by gel electrophoresis using a NuPage 4-12% Bis-Tris pre-cast gel (Invitrogen) in MES buffer (Invitrogen). The proteins were electrotransferred to a PVDF membrane (Macherey-Nagel, Düren, Germany). The membrane was blocked in 5% dry milk in TBS-T for 1 hour at RT and was then incubated with primary antibodies at 4°C overnight. After washing the membrane three times, proteins were visualized by enhanced chemiluminescence according to the manufacturer´s instructions (ECL Plus and ECL Prime; GE Healthcare, Munich, Germany). The signals were captured using a FluoroChem Q (Biozym Scientific GmbH, Hessisch Oldendorf, Germany) and analyzed using FluoroChem Q SA Software (Biozym Scientific GmbH).

### Reverse transcription and quantitative RT-PCR

Total RNA from cells was extracted using TriReagent (Sigma). RNA integrity and quantity were assessed by agarose gel electrophoresis and spectrophotometry, respectively. Subsequently, 1 μg of total RNA was reverse transcribed using Oligo dT primers and the Superscript Plus Kit (both from Invitrogen) according to the manufacturer´s instructions. The quantification of receptor expression and the expression of the two housekeeping genes *porphobilinogen deaminase* (PBGD) and *TATA box binding protein* (TBP) was determined using the 7900HT sequence detection system (Applied Biosystems, Darmstadt, Germany) using the SYBR-Green chemistry kit (Qiagen, Hilden, Germany). The 5-μl reaction from the kit was supplemented with 2.5 μl of cDNA (diluted 1:20) and 0.25 μM of gene-specific primers for PDGFRβ, c-KIT, FLT3, MCSF, PBGD and TBP, respectively. All primers (Operon, Cologne, Germany) were designed using the primer3 on-line primer design program (http://www-genome.wi.mit.edu/genome_software/other/primer3.html.). Primers used for quantitative RT-PCR are listed in the supplemental information section of the current manuscript. A standard curve for quantitative PCR was generated with the same reaction set-up using one control sample as a standard (1:4 to 1:80). Fluorescence signals were monitored using the 7900HT sequence detection system and terminated when all reactions reached an amplification plateau, whereas a template-free control remained at a basal level. Data analysis was performed using the detection system software SDS 2.1 (Applied Biosystems). To verify that only specific PCR products evoked fluorescence signals, PCR products were run on 2% agarose gels and analyzed using the E.A.S.Y. Win 32 software (Herolab, Wiesloch, Germany). Receptor mRNA expression was normalized to both PBGD and TBP mRNA expression, respectively, to compensate for different sample capacities. All quantification assays were performed in triplicate.

### Transfection

CRC cells were plated in 12-well plates at a density of 6.5 × 10^4^ cells per well before transfection with siRNA oligonucleotides. After an incubation of 24 h, the cells were transfected using Oligofectamine Reagent and OPTIMEM I medium (both from Invitrogen, Karlsruhe, Germany) according to the supplier's instructions with different IGF-IR or EGFR gene-specific stealth™ siRNA duplex oligonucleotides (Invitrogen) at a final concentration of 80 nM, respectively. As a control, cells were transfected with siRNA duplex oligonucleotides (Eurogentec, Seraing, Belgium) against the firefly (*Photinus pyralis*) luciferase gene (Luc). Forty-eight and seventy-two hours after transfection, cells were collected and used in the following experiments. The target sequences of the gene-specific stealth™ siRNA were as follows:

Luciferase (LUC) 5'- CGUACGCGGAAUACUUCGATT-3'
Human PDGFR β 37 (P37) 5'-UGUAGAGGCUGUUGAAGAUGCUCUC-3'
Human PDGFR β 58 (P58) 5'-UUCCCGAUCACAAUGCACAUGAGGG-3'
Human PDGFR β 60 (P60) 5'-CGGAAGCAGAGGAUAGCUUCCUGUA-3'
Human c-Kit 20 (cK20) 5'-CCUGCAGCGAUAGUACUAAUGAGUA-3'
Human c-Kit 21 (cK21) 5'-CAUGGACAUGAAACCUGGAGUUUCU-3'
Human c-Kit 22 (cK22) 5'-CCAGAGACAUCAAGAAUGAUUCUAA-3'
Human c-Kit 6 (cK6) 5'-CTCGCACCTTTCCAAAGTTAA-3'

### Determination of cell proliferation and cytotoxicity

After transfection with siRNA oligonucleotides or treatment with the accordant inhibitors, cells (5 × 10^3^ cells/well) were plated in 100 μl of cell culture medium in a 96-well plate as described above. After different periods of incubation (3, 24, 48 and 72 h), cell proliferation was determined using the CellTiter 96^®^ AQ_ueous_ Non-Radioactive Cell Proliferation Assay (MTS; Promega, Mannheim, Germany) according to the manufacturer's instructions. A microplate reader (E800x; BioTek Instruments, Inc., Winooski, Vermont) was used to measure the optical density at a wavelength of 495 nm and a reference wavelength of 620 nm. All experiments were performed in triplicate. For measuring cytotoxicity, the LDH Plus Cytotoxicity Kit (Roche) was used according to the manufacturer´s instructions.

### Cell cycle analyses

After transfection with siRNA or treatment with inhibitor, cells were harvested and fixed in ice-cold 70% ethanol for at least 2 hours. For propidium-iodide staining, cells were centrifuged and resuspended in 10 μg/ml propidium-iodide with 100 μg/ml RNase A. After incubation for 30 minutes at 37°C, flow cytometric measurements were performed using a FACSCalibur flow cytometer (BD Biosciences, Heidelberg, Germany) and analyzed using the CellQuestPro software (BD Biosciences).

### PathScan RTK signaling antibody array

To analyze receptor activation, the PathScan RTK signaling antibody array (Cell Signaling) was used according to the manufacturer´s instructions. Briefly, SW480 cells were transfected with siRNAs against luciferase gene, PDGFRβ (siRNA P37), c-KIT (siRNA cK6) or both PDGFRβ and c-KIT and were then incubated for 48 hours. After serum starvation overnight, cells were stimulated with 10% FCS for 10 minutes, and the protein was isolated. For treatment with the inhibitor, SW480 cells were incubated in the presence of 1 μM and 5 μM Ki11502 for 48 h, respectively, serum-starved overnight and stimulated with 10% FCS according to the siRNA samples. Next, 45 μg of protein at a concentration of 0.3 mg/ml was hybridized to the antibody array. Fluorescent readout was performed on a Fluorochem Q (Biozym). Data analyses were carried out using the Fluorochem Q SA software.

### Statistical analysis

All experiments were replicated three or four times. Autoradiographs of western blots were scanned (BIO-RAD, Hercules, CA, USA). After background subtraction, the densitometry of individual bands was analyzed using the ImageJ software (Version 1.34 s, NIH, Bethesda) according to the manufacturer's instructions. The relative densities of the bands were expressed as a percentage of the control. Proliferation assays, cell viability experiments and apoptosis studies were each performed in triplicate. The means ± standard deviation (SD) were indicated relative to the control. Student's *t*-test for paired values was used, and a *P* value less than 0.05 was deemed to be statistically significant.

## SUPPLEMENTARY FIGURES


