# Supplementary material for: Blockade of the PDGFR family together with SRC leads to diminished proliferation of colorectal cancer cells
Source: Oncotarget. 2013 Jun 30;4(7):1037–49. doi: 10.18632/oncotarget.1085 (PMC3759664; doi:10.18632/oncotarget.1085)
Supplement: Supplementary file 1 [file oncotarget-04-1037-s001.pdf]

Blockade of the PDGFR family together with SRC leads to diminished proliferation of colorectal cancer cells - Kaulfuß et al

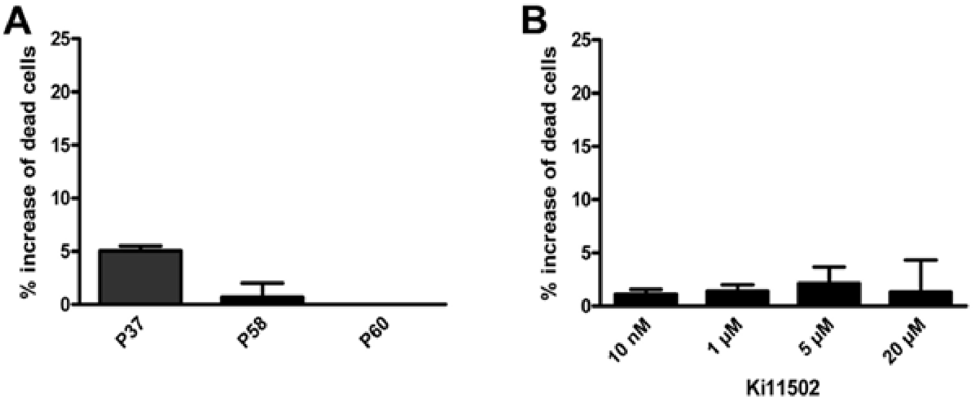

Supplementary Figure 1: Effect of PDGFRβ blockade on apoptosis of SW480 cells. (a) SW480 cells were transfected with PDGFRβ-specific siRNAs (P37, P58, P60) or luciferase (Luc) siRNA as indicated. After transfection, SW480 cells were plated in a 96-well plate, and cell viability was evaluated using the MultiTox-Fluor Multiplex Cytotoxicity Assay 72 h after transfection. The number of dead cells in Luc-transfected SW480 cells was defined as the basal level. An increase of dead cells after PDGFRβ-siRNA transfection was indicated as a percent increase over the basal level of Luc-transfected SW480 cells. (b) After starvation of SW480 cells for 24 h, cells were maintained in the presence or absence of the PDGFRβ inhibitor Ki11502. Control cultures were maintained in serum-reduced medium. All cells received the same amount of DMSO. Cell viability was evaluated using the MultiTox-Fluor Multiplex Cytotoxicity Assay. An increase of dead cells after treatment with Ki11502 was indicated as a percent increase over the basal level of control SW480 cells. Each bar represents the mean ± SD of triplicate values from two experiments.

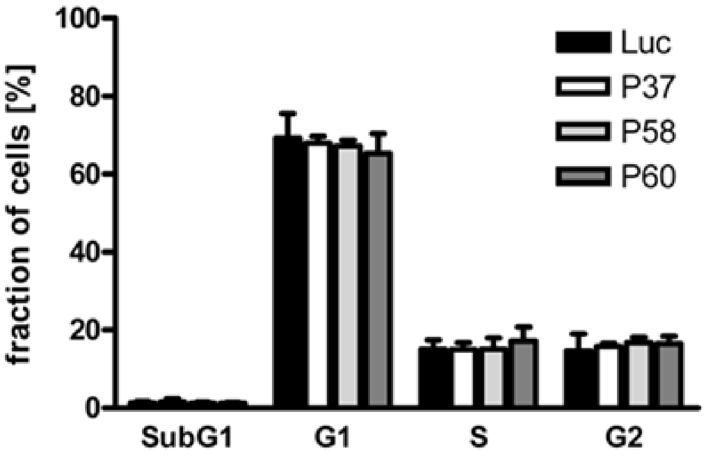

Supplementary Figure 2: Effect of PDGFRβ blockade on cell cycle distribution using siRNA. SW480 cells were transfected with PDGFRβ-specific siRNAs (P37, P58, P60) or luciferase (Luc) siRNA as indicated. After transfection, SW480 cells were subjected to flow cytometry. No difference between cell cycle distributions was observed after PDGFRβ knockdown. Each bar represents the mean ± SD of triplicate values from two independent experiments.

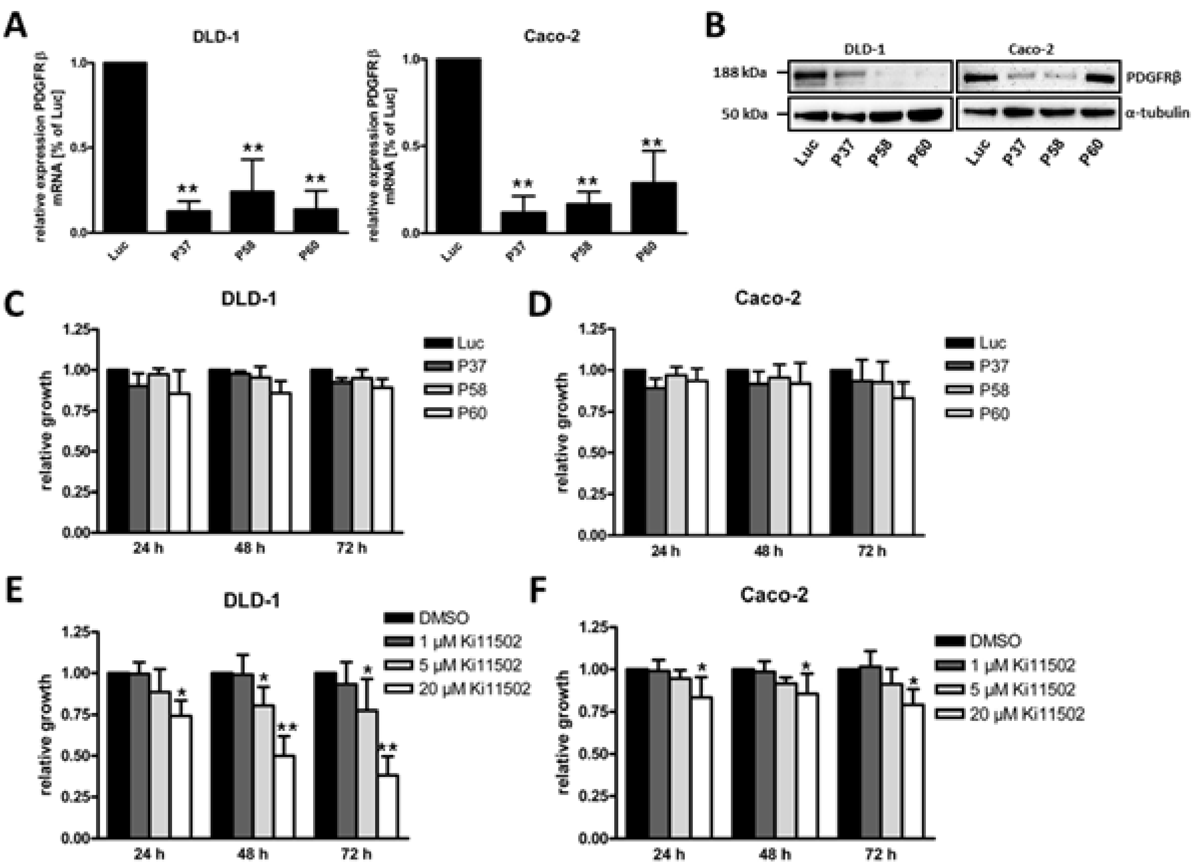

Supplementary Figure 3: Effect of PDGFRβ blockade on DLD-1 and Caco-2 cells. DLD-1 and Caco-2 cells were transfected with siRNAs against PDGFRβ (P37, P58 and P60) and luciferase (Luc). (A, B) Knockdown of PDGFRβ expression was verified using quantitative RT-PCR (A) and western blotting (B) after 48 h. (C, D) After transfection of PDGFRβ-specific siRNAs, proliferation assays were performed. (E, F) DLD-1 and Caco-2 cells were treated with the PDGFRβ inhibitor Ki11502, and cell proliferation was determined after 24, 48 and 72 h. Whereas PDGFRβ knockdown using siRNAs resulted only in a moderate decrease of proliferation in DLD-1 cells, inhibitor treatment with Ki11502 diminished the proliferation time and was dose dependent. Caco-2 cells did not respond to PDGFRβ blockade. The results are expressed as the percent decrease (mean ± SD of three independent experiments) compared with Luc siRNA-transfected and vehicle-treated cells. Statistically significant differences relative to Luc siRNA-transfected cells are indicated: *, $P < 0.05$; **, $P < 0.01$ (Student's t-test).

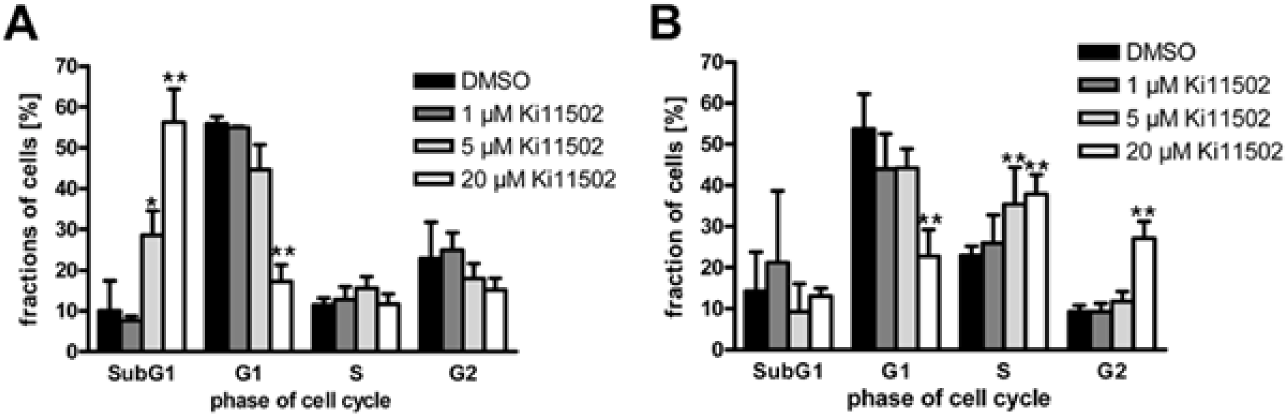

Supplementary Figure 4: Effect of PDGFRβ inhibition with Ki11502 on cell cycle distribution in DLD-1 (A) and Caco-2 (B) cells. Cells were treated with the PDGFRβ inhibitor Ki11502 for 72 h and were then subjected to flow cytometric analyses. Bars represent the proportion of cells in the indicated cell cycle of three independent experiments: bars, SD. *, $P < 0.05$; **, $P <0.01$, statistically significant differences relative to CRC cells treated with DMSO (Student's t-test).

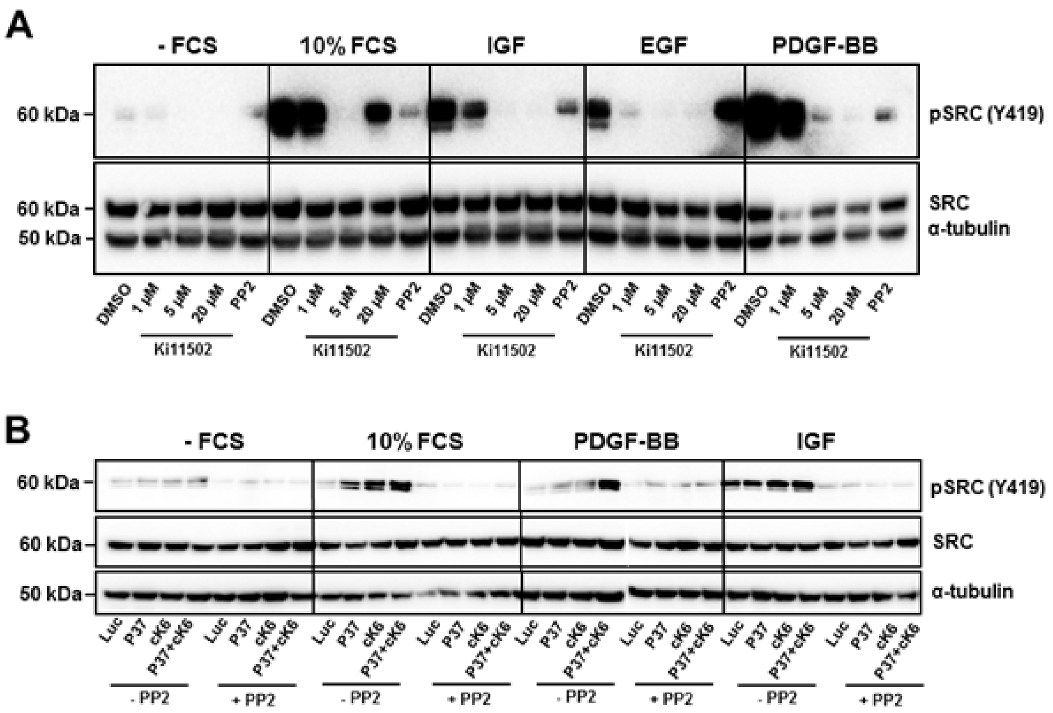

Supplementary Figure 5: SRC activation after PDGFRβ inhibition using siRNA or the small-molecule inhibitor Ki11502 in DLD-1 cells. (A) DLD-1 cells were treated with Ki11502 (1, 5 and 20 µM) or with 10 µM of the SRC inhibitor PP2 for 48 h. After serum starvation, cells were stimulated with 10% FCS, PDGF-BB, EGF and IGF for 10 minutes. (B) DLD-1 cells were transfected with siRNA against PDGFRβ (P37), c-KIT (cK6) or both (P37+cK6) compared with control-transfected cells (Luc) and were then treated with or without 10 µM PP2 for 48 h. After serum starvation, cells were stimulated with 10% FCS, PDGF-BB, EGF and IGF for 10 minutes. Whereas inhibitor treatment resulted in a reduction of SRC activation (A), the down-regulation of PDGFRβ, c-KIT or both did not change the SRC activation pattern (B).

Primer sequences for quantitative PCR

Platelet-derived growth factor receptor β

hPDGFRB-Q1-Fw	5‘-AGCATCTTCAACAGCCTCTACACC-3‘
hPDGFRB-Q1-Rev	5‘-TCAAACTTCTCTTCCCAGCACTTC-3‘
PDGFRB-Q6-FW	5‘-CAGCAATGCTCTGCCCGTTGGGCT-3‘
PDGFRB-Q6-Rev	5‘-GCAGGTCCTCTCAGGGGCAGAGGG-3‘

v-kit Hardy-Zuckerman 4 feline sarcoma viral oncogene homolog

(c-Kit) receptor

C-KIT-Q4-FW	5‘-TGCACTTGGGCGAGAGCTGGAACG-3‘
C-KIT-Q4-REV	5‘-ACAGCCTAATCTCGTCGCCCACGC-3‘
C-KIT-Q5-FW	5‘-CTCGCGGCGCCTGGGATTTTCTCT-3‘
C-KIT-Q5-REV	5´-CTAATCTCGTCGCCCACGCGGACT-3´
C-KIT-Q7-FW	5´-CCGAAGGAGGCACTTACACATTCCT-3´
C-KIT-Q7-RW	5´-AGAGCATCTCTGCTCAGTTCCTGGAC-3´
C-KIT-Q11-FW	5´-CTCTGCTTCTGTACTGCCAGTGGATG-3´
C-KIT-Q11-RW	5´-AACTCAGCCTGTTCCTGGGAAACTCC-3´
FLT3-Q1-Fw	5´-CCAGCTGCCGCTGCTCGTTGTTTTT-3´
FLT3-Q1-Rev	5´-TCTGGGGTCTCAACGCACACCCGA-3´
MCSF-Q1-Fw	5´-TGGGCAATGGCAGCGTGGAATGGG-3´
MCSF-Q1-Rev	5´-AGGGCCGGGCAGGGTCTTTGACAT-3´
